# Glycoproteomics revealed novel N-glycosylation biomarkers for early diagnosis of lung adenocarcinoma cancers

**DOI:** 10.1186/s12014-022-09376-8

**Published:** 2022-11-19

**Authors:** Kai Fang, Qin Long, Zhonghua Liao, Chaoyu Zhang, Zhiqiang Jiang

**Affiliations:** 1 Department of Emergency, The Second Affiliated Hospital of Chengdu Medical College, China National Nuclear Corporation 416 Hospital, Chengdu, 610051 China; 2 Department of Thyroid Surgery, The Second Affiliated Hospital of Chengdu Medical College, China National Nuclear Corporation 416 Hospital, Chengdu, 610051 People’s Republic of China

**Keywords:** Lung adenocarcinoma, Early diagnosis, Circulating biomarkers, N-glycosylation, Machine learning

## Abstract

Circulating biomarkers play important roles in diagnosis of malignant tumors. N-glycosylation is an important post-translation patter and obviously affect biological behaviors of malignant tumor cells. However, the role of N-glycosylation sites in early diagnosis of tumors still remains further investigation. In this study, plasma from 20 lung adenocarcinoma (LUAD), which were all classified as stage I, as well as 20 normal controls (NL) were labeled and screened by mass spectrometry (MS). Total 39 differential N-glycosylation sites were detected in LUAD, 17 were up-regulated and 22 were down-regulated. In all differential sites, ITGB3-680 showed highest potential in LUAD which showed 99.2% AUC, 95.0% SP and 95.0% SN. Besides, APOB-1523 (AUC: 89.0%, SP: 95.0%, SN: 70.0%), APOB-2982 (AUC: 86.8%, SP: 95.0%, SN: 45.0%) and LPAL2-101 (AUC: 81.1%, SP: 95.0%, SN: 47.4%) also acted as candidate biomarkers in LUAD. Combination analysis was then performed by random forest model, all samples were divided into training group (16 cases) and testing group (4 cases) and conducted by feature selection, machine learning, integrated model of classifier and model evaluation. And the results indicated that combination of differential sites could reach 100% AUC in both training and testing group. Taken together, our study revealed multiple N-glycosylation sites which could be applied as candidate biomarkers for early diagnosis diagnosis of LUAD.

**Supplementary Information:**

The online version contains supplementary material available at 10.1186/s12014-022-09376-8.

## Introduction

Lung cancer (LC) is the leading cause of malignant cancer-related mortality worldwide. Non-small cell lung cancer (NSCLC) and small cell lung cancer (SCLC) are two subtypes of lung cancer, in which NSCLC accounts for 85% of lung cancer cases [1]. NSCLC can also be divided as lung adenocarcinoma (LUAD) (50%), squamous carcinoma (LUSC) (35%) and large cell carcinoma (15%) according to histologic differentiation [2]. Despite clinical application of advanced therapeutic strategies, including target and radiation therapies, the 5-year survival rate of lung cancer still remain in less than 20% [3], which mainly due to late diagnosis [4]. Early diagnosis can contribute greatly to survival rate of LC patients, which is now completed by low-dose computed tomography (CT) and promotes early diagnosis of LC by detecting small malignant nodule [5], but high-false positive rate, as well as radiation injury and high financial burden are points of controversy [6]. Circulating biomarkers from blood, bronchoalveolar lavage fluid and sputum, which consist by proteins, exosomes, miRNA and circulating free DNA (cfDNA), can be helpful in detecting early lung cancer due by noninvasive, convenient and inexpensive acquisitions [7]. Clinical application biomarkers in lung cancer include carcinoembryonic antigen (CEA) [8], carbohydrate antigen 19–9 (CA199) [9], carbohydrate antigen 12–5 (CA125) [10], LUSC specific marker Cyfra21-1 [11] and SCLC specific marker (NSE) [12]. However, these markers is only valuable in advanced stages (III + IV) and is poorly sensitive in early stage (I) of lung cancer, so selecting and identification of novel biomarkers for early diagnosis of lung cancer is very important.

Recently studies reveal novel circulating molecules which can be used as candidate biomarkers in early diagnosis of lung cancer, including circulating tumor DNA (ctDNA) [13], exosomes [14], circulating tumor cells (CTC) [15] and autoantibodies [16]. Among these molecules, exosomes are double-lipid and nanosized (30–150 nm diameter) vesicles secreted by almost all cell types, which are released into extracellular microenvironment [17] and participate in tumor metastases by establishing microenvironment via exchange oncogenic molecules with nearby and distant cells [18]. Based on the characteristics exosomes, the contents in exosomes, include proteins, lipids, nucleic acid and metabolites, can be conducted as valuablebiomarkers for tumor diagnosis [19]. Autoantibodies (AAbs) are tumor immune-related markers for malignant tumors, produced by immune response against neo-antigens formed by mutated genes and aberrantly expressed proteins [20]. Although rapid degradation and clearance in serum levels limits their clinical application, AAbs assay still act as a promising method in distinguishing normal individuals and non-malignant benign disease.

Besides above tumor-related molecules, glycoproteomic technologies, based on mass spectrometry, has now been applied in discovery of biomarker for diagnosis of malignant tumors [21]. In this study, we detected glycoproteomic levels in 20 LUAD patients, which were all classified as stage I. We also conducted 20 healthy controls (NL) to screen novel glycoprotein biomarkers for early diagnosis of LAUD.

## Materials and methods

### Participants and samples

This study contained two groups, LUAD patients were from the Department of Respiration and Thoracic Surgery who had not obtained surgery or other treatment such as chemical, target or radiation therapy. All clinical characteristics, including age, gender and TNM stage were collected by medical records. All healthy samples were from the Center of Health Management, participants were exclude if their family had any history of cancer and inflammation. Plasma samples were extracted and stored in − 80 °C before applied for detection. This study was approved by Medical Ethics Committee and Institutional review board of China National Nuclear Corporation 416 Hospital, with all participants providing written informed consents. Plasma samples were extracted and stored in − 80 °C according to standard procedures.

### Protein extraction and TMT labeling

The supernatants of plasma samples were centrifugated at 12000 g at 4 °C for 10 min to remove cellular debris and then transferred to a new 1.5 ml tube. Protein concentration was then detected by BCA kit in accordance to the manufacturer’s manual. All collected plasma samples were digested by treating with 5 mM dithiothreitol (56 °C, 30 min) and alkylated with 11 mM iodoacetamide (Room temperature, 30 min in darkness). TEAB (100 mM) was added to dilute samples to urea concentration less than 2 M. For protein digestion, trypsin was added at 1:50 mass ration (trypsin-to-protein) overnight for first digestion and 1:100 ratio mass for second digestion (4 h).

Strata XC18 SPE column (Phenomenex) and vaccum-dired was used to desalt digested peptides and then reconstituted in 0.5 M TEAB and processed by TMT 10plex Mass Tag Labeling kit according to manual instruction.

### HPLC fractionation, affinity enrichment

HILIC enrichment was conducted to N-glycosylaiton modification. Tryptic peptides were firstly re-dissolved in 200 μL washing buffer (80% CAN, 5% TFA) and then washed with washing buffer three times after loading into the column. TFA (0.1%), ammonium bicarbonate (50 mM) and CAN (50%) were added to elute glycopeptides two times, which were then dried in Speedvac and re-dissovled with 50 μL ammonium bicarbonate solution (50 mM). The digestion was completed at 37 °C overnight by adding 2 μL PNGase F glycodidase. Finally all de-glycopeptides were desalted by C18 Zip Tips according to manual instruction and dried for further MS analysis.

### LC–MS/MS analysis

ALL tryptic peptides were dissolved in solvent A (0.1% formic acid, 2% acetonitrile in water) and loaded into reverse-phase analytical column, and then separated by solvent B (0.1% formic acid in 90% acetonitrile) with gradient 4%-20% for 38 min, 20%-32% within 16 min, climbing to 80% in 3 min, holding in 80% for last 3 min, all at a constant flowrate of 500nL/min on an EASY-nLC 1200 UPLC system (Thermo Fisher Scientific). Q ExactiveTM HF-X (Thermo Fisher Scientific) with a nano-electrospray ion source was used to analyze all separated peptides, with 2.1 kV electrospray voltage. The full MS scan resolution was set to 120,000 for a scan range of 350–1400 m/z and then up to 20 most abundant precursors were then selected for further MS/MS analyses with 15 s dynamic exclusion. The HCD fragmentation was processed in normalized collision energy (NCE) (28%) and detected in Orbitrap (resolution: 30,000) with 100 m/z fixed first mass. Automatic gain control (AGC) target was set at 1E5, with an intensity threshold of 1E5 and a maximum injection time of 100 ms. The TMT LC–MS/MS analysis in our research was supported by PTM BioLabs.

### Database search

Raw data form LC–MS/MS was processed by Maxquant search engine (v.1.5.2.8). When searching the library, variable modification adds oxidation of methionine, acetylation of the N-terminal of the protein, deamidation (NQ), and deamidation of asparagine (18O), by detecting the mass deviation on asparagine to confirm whether N-glycosylation occurred. Human uniprot database, together with reverse decoy database were used to search tandem mass spectra. The mass tolerance for precursor ions was 20 ppm and 5 ppm in first research and main search respectively, while the mass tolerance for fragment ions was set as 0.02 Da. Carbamidomethyl on Cys was specified as fixed and acetylation modification, and the oxidation on Met were specified as variable modifications. The resulting MS/MS data were processed using Proteome Discoverer (v2.4.1.15). Tandem mass spectra were searched against the Uniprot_Homo_sapiens_9606_20210721 database (78,120 entries) concatenated with reverse decoy database. Trypsin (Full) was specified as cleavage enzyme allowing up to 2 missing cleavages; the minimum length of the peptide segment is set to 6 amino acid residues; the maximum number of peptide modifications is set to 3; the mass tolerance for precursor ions was set as 20 ppm, and the mass tolerance for fragment ions was set as 0.02 Da. Carbamidomethyl (C), TMT6plex (peptide N-Terminus), TMT6plex (K) were set as fixed modifications, and Acetyl (protein N-Terminus), Oxidation (M), Deamidated: 18O (N) were set as variable modifications. The quantitative method was set to TMT-11plex, and the FDR of protein identification and PSM identification were both set to 1%.

### GO and domain annotation

UniProt-GOA database ( http://www.ebi.ac.uk/GOA/) was used in Gene Ontology (GO) annotation proteome and identified protein ID was converted and mapped to UniProt ID and GO ID. Based on protein sequence not annotated by UniProt-GOA database, the InterProScan soft was applied to annotated protein’s GO functional. All proteins were defined by Gene Ontology annotation based on three categories: biological process, cellular component and molecular function. Proteins domains annotated by InterProScan were identified InterPro domain database based on protein sequence alignment method.

### KEGG pathway annotation and subcellular localization

Kyoto Encyclopedia of Genes and Genomes (KEGG) was a networks which connected all known molecular interaction, including genes and proteins, pathways and complexes, which also contained biochemical compounds and reactions. The contents of KEGG pathway included metabolism, genetic process information, cellular processes and drug development. KEGG online service tools KAAs was firstly introduced to annotate the protein description, and then another online service tools KEGG mapper was conducted to mapping the annotation results. Wolfpsort was an updated version of PSORT/PSORT II for the prediction of eukaryotic sequences, which was introduced in this study to predict subcellular localization. CELLO was conducted to subcellular localization prediction.

### Motif analysis

The model of sequences was identified by Soft MoMo (motif-x algorithm), which constituted with amino acids in specific positions of modify-21-mers (10 amino acids upstream and downstream of the site) in all protein sequences. And all the database protein sequences were used as background database parameter. Minimum number of occurrences was set to 20 and emulate original motif-x was ticked, and other parameters with default.

### Functional of gene ontology enrichment

All identified proteins was separated into three categories by GO annotation: biological process, cellular compartment and molecular function. For each category, the enrichment of the differentially modified protein against all identified proteins and pathway enrichment were both identified by two-tailed Fisher’s exact test. These pathways were classified into hierarchical categories according to KEGG website. For all analysis, p-value < 0.05 was considered significant.

### Enrichment-based clustering

TO further hierarchical cluster based on differentially modified protein functional classification (such as: GO, Domain, Pathway, Complex), we collected all available categories after enrichment along with P values and filtered for those categories which were at least enriched in one of the clusters with P value < 0.05. This filtered P value matrix was transformed by the function x =  − log10. All x values were z-transformed for each functional category and z scores were clustered by one-way hierarchical clusteringin Genesis and the cluster membership were visualized by “heatmap.2” function from the “gplots” R-package.

### Protein-proteins interaction network

All differentiated proteins were searched in STRING database (version 10.0) to investigate protein–protein interactions. Only interactions between the proteins belonging to the searched data set were selected, STRING defines a metric called “confidence score” to define interaction confidence and all interactions had a confidence score ≥ 0.7 (high confidence). Interaction network form STRING was visualized in R package “networkD3”.

## Results

### Study design and clinical characterizations of all patients

This study contained two groups, including 20 LUAD patients and 20 healthy controls (NL). The average age was 57.8 ± 10.8 and 45.1 ± 11.4 in LUAD and NL groups, and LUAD included 6 male and 14 female, while NL contained 7 male and 13 female. All LUAD patients were classified as stage I according to 7th version, and 7 had smoke history while 13 were never smoking. All clinical features of all participants were listed in Table 1.

**Table 1:** The clinical features of all participates in this study

Number	Sex	Age	Smoke history	TNM	Stage
LA1	Female	75	No	T1bN0M0	IA2
LA2	Male	61	Yes	T1bN0M0	IA2
LA3	Female	37	No	T1aN0M0	IA1
LA4	Male	63	Yes	T1cN0M0	IA3
LA5	Female	62	No	T1bN0M0	IA2
LA6	Male	61	Yes	T1c N0M0	IA3
LA7	Female	57	No	T2aN0M0	IB
LA8	Female	51	No	T1bN0M0	IA2
LA9	Male	51	No	T1bN0M0	IA2
LA10	Female	46	No	T1b/2aN0M0	IA2/
LA11	Female	49	No	T1cN0M0	IA3
LA12	Female	42	No	T1bN0M0	IA2
LA13	Female	60	No	T1aN0M0	IA1
LA14	Female	57	No	T2aN0M0	IB
LA15	Male	70	Yes	T1bN0M0	IA2
LA16	Male	82	No	T2aN0M0	IB
LA17	Female	65	No	T1bN0M0	IA2
LA18	Female	59	No	T1bN0M0	IA2
LA19	Female	63	No	T2aN0M0	IB
LA20	Female	45	No	PT1bN0M0	IA2

Number	Sex	Age
NL1	Female	31
NL2	Female	71
NL3	Female	40
NL4	Female	41
NL5	Female	41
NL6	Female	40
NL7	Male	41
NL8	Male	50
NL9	Female	29
NL10	Female	58
NL11	Male	31
NL12	Female	49
NL13	Female	48
NL14	Male	45
NL15	Male	51
NL16	Male	49
NL17	Female	38
NL18	Female	71
NL19	Female	47
NL20	Male	31

**Fig. 1:**
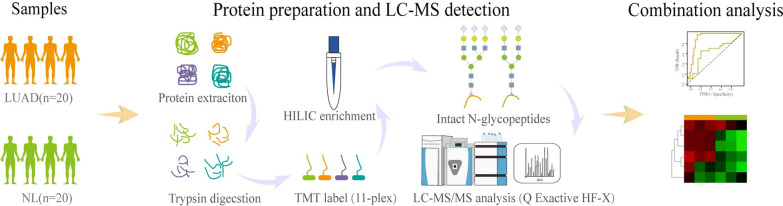
Workflow of N-glycosylation analysis in LUAD patients and NL controls. Trypsin was added into all samples for protein digestion and then processed by TMT kit/iTRAQ kit. High pH reverse-phase HPLC was performed to fractionate tryptic peptides and then dissolved in NETN buffer for enrichment. The peptides were then subjected to tandem mass spectrometry (LC–MS/MS) in Q ExactiveTM Plus. A data-dependent procedure was then conducted to peptides and alternated between one MS scan followed by 20 LC–MS/MS scans with 15.0 s dynamic exclusion

To explore N-glycoprotein sites for lung cancer diagnosis, plasma were obtained from LUAD and NL controls and were extracted and labelled. After fractionation and enrichment, LC–MS/MS was performed to investigate the N-glycoprotein levels in all samples. The differential N-glycoprotein levels between LUAD and NL were analyzed based on databases. And the candidate N-glycoprotein sites were combined to study the role of novel biomarkers for future lung cancer diagnosis. The study procedures were listed in Fig. 1.

### Characteristics of the identified N-glycoproteins

**Fig. 2:**
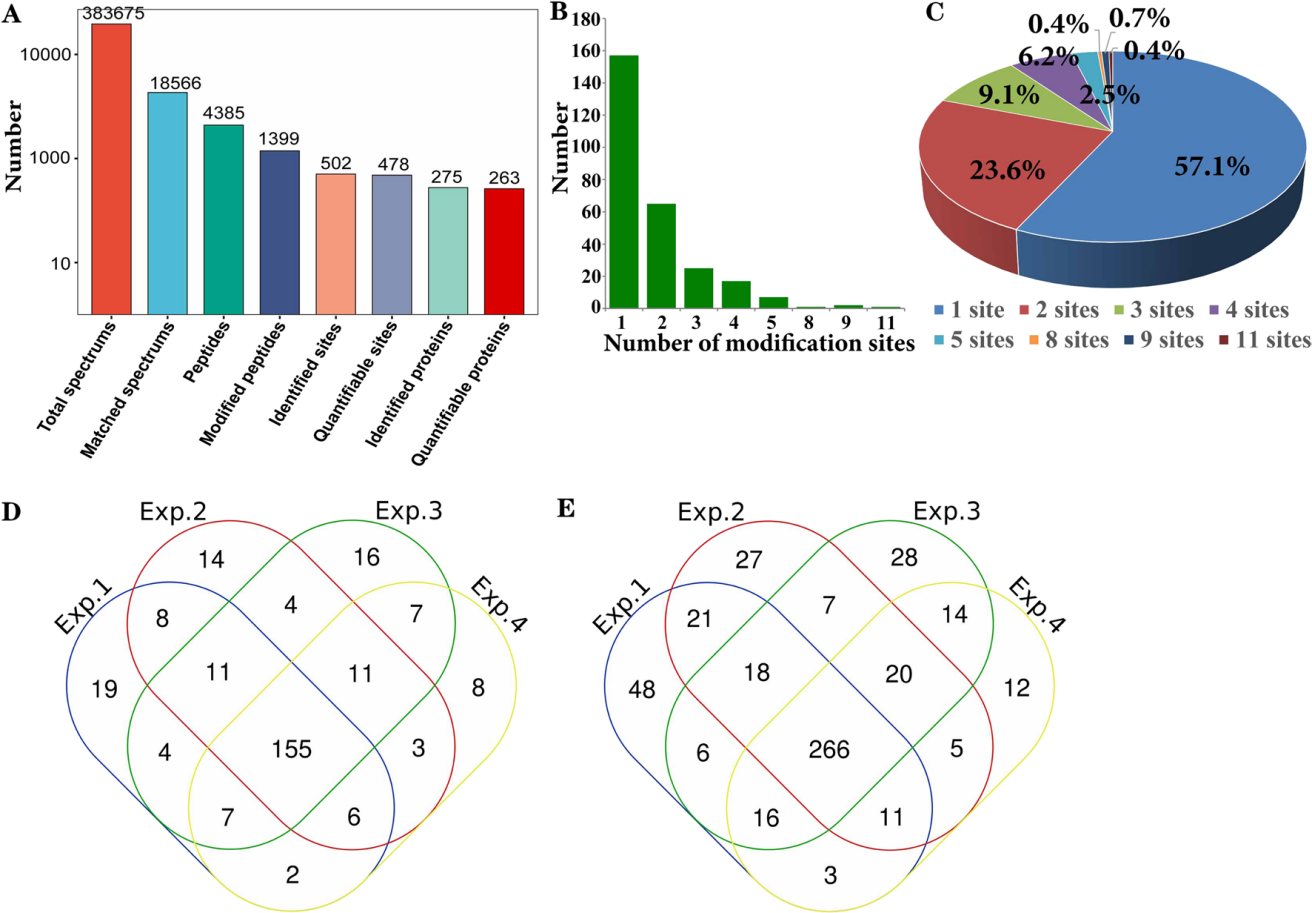
Characteristics of identified N-glycoproteins. **A** Total number of identified N-glycosylation proteins. **B** The number of modification sites per protein. **C** Pie chart showed the number and proportions of single and multiple N-glycosylated sites in LUAD. **D** Overlap numbers of glycoproteins in LUAD samples. E Overlap numbers of glycosylation sites in LUAD samples

Next, we performed enriched peptides and detected by LC–MS/MS to identify N-glycoproteins in obtained plasma samples. In comparison to NL samples, we obtained total 383,675 spectrums in LUAD patients and 18,566 matched spectrums were identified based on protein datasets (Additional file 1: Table S1). We then identified total 4385 peptides in matched spectrums. In all peptides, 1399 were belongs to modified peptides which contained 502 identified sites. In LUAD samples, 478 sites were quantified which were identified from 275 proteins (Fig. 2A).

In this study we identified total 478 sites in 263 proteins in LUAD patients. The number of N-glycosylated sites assigned to all proteins ranged from 1 to 11 with average degree of glycosylation was 2.5. More than half of glycoproteins (270/478, 57.1%) carried only a single N-glycoprotein site, 110 (23.6%) of them harbored double N-glycoprotein sites (Fig. 2B, C). Triple and four N-glycoprotein sites were 41 (9.1%) and 27 (6.2%), respectively. And the rest 17 (4%) contained five (8, 2.5%), eight (1, 0.4%), nine (3, 0.7%) and eleven (1, 0.4%) sites (Fig. 2B, C). And our results also indicated the overlap numbers in all samples in both protein and N-glycoprotein sites (Fig. 2D, E). In summary, multiple proteins and N-glycosylation sites were identified in LUAD patients, which could be conducted to further analysis to identify candidate biomarkers for future clinical application.

### Disease-associated changes in N-glycopeptide abundance in LUAD

**Fig. 3:**
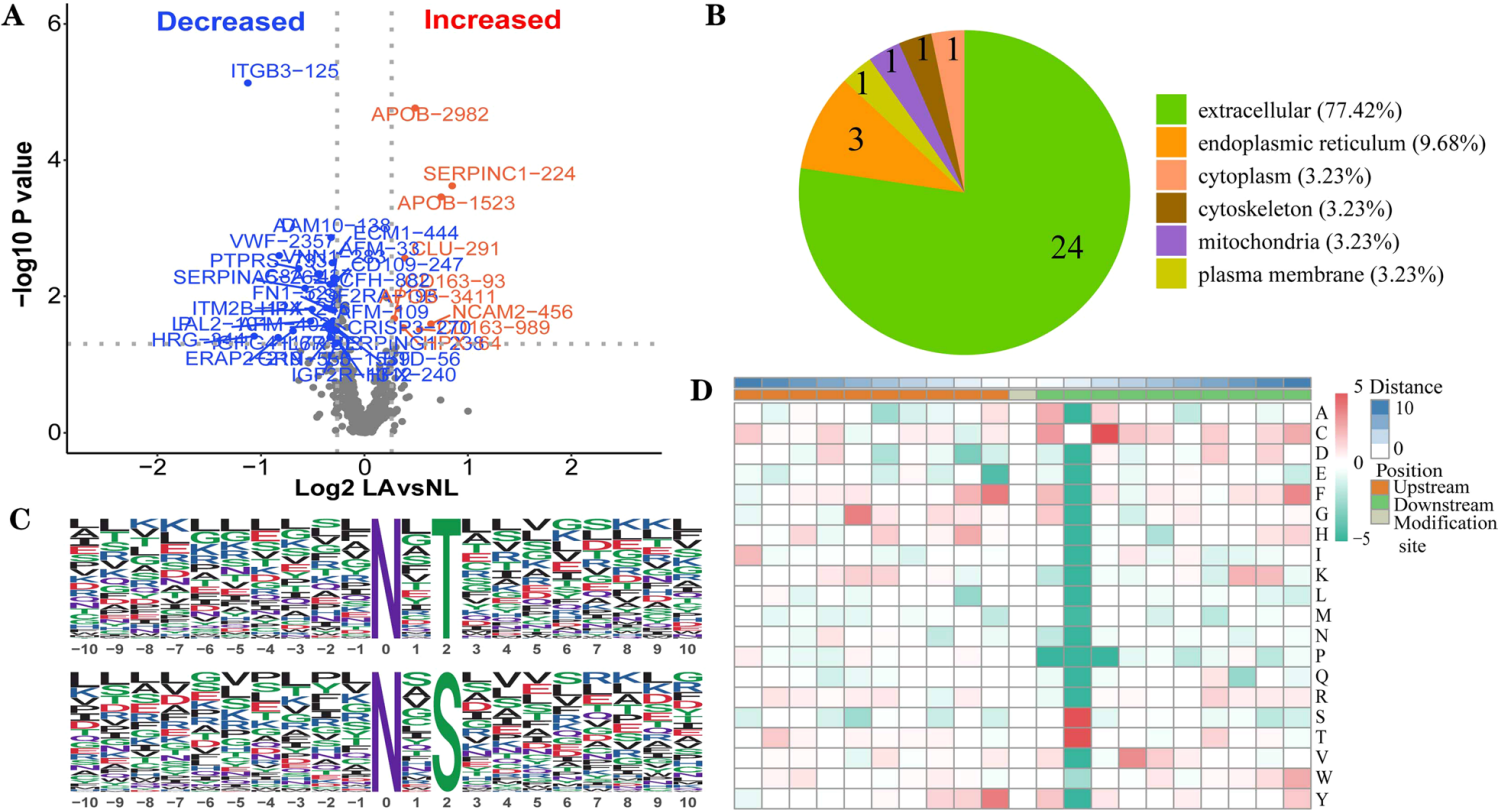
Disease-associated variations in N-glycopeptide abundance in LAUD. **A** Volcano revealed increased and decreased N-glycosylation sites in LUAD and NL samples. **B** Classification of identified N-glycosylation proteins based on subcellular loction. **C** Sequence motifs located nearby the target asparagine in enriched glycosylation sites. **D** Heatmap showing the relative frequency of amino acids in the proximity of asparaine (enrichment, red; depletion, green)

In comparison to NL samples, 39 differential N-glycosylation sites were obtained in LUAD. In all differential sites, 17 increased in LUAD patients, such as APOB-2982, SERPINC1-224 and APOB-1523, while 22 decreased in lung cancer samples, including ITGB3-125 and VWF-235 (Fig. 3A). We analyzed the cellular distribution of the differential proteins, and the result indicated 24 proteins were extracellular, 3 proteins were endoplasmic reticulum, while the cytoplasm, cytoskeletio, mitochondria and plasma membrane contained 1 protein (Fig. 3B).

The neighborhood residues of glycosylated asparagines could determine the specificity of LUAD. MoMo was conducted to obtain the characteristic sequence of modified sites and their enrichment statistics. As shown in Fig. 3C, 2 conserved amino acids flanking the glycosylated asparagine residues (from − 10 to + 10) were defined. These motifs included N-x-T-*-Y and N-x-S, where x represented any amino acid except proline and the asterisk denoted a random amino acid. Based on analysis of hierarchical clusters, threonine and serine displayed the highest probability at the position + 2, while the frequency of a proline residue in the proximity was markedly underrepresented (Fig. 3D). Taken together, our results suggested a preference motif exposed to the surface of glycoproteins.

### Analysis and annotation of differentially N-glycosylated proteins in LUAD

**Fig. 4:**
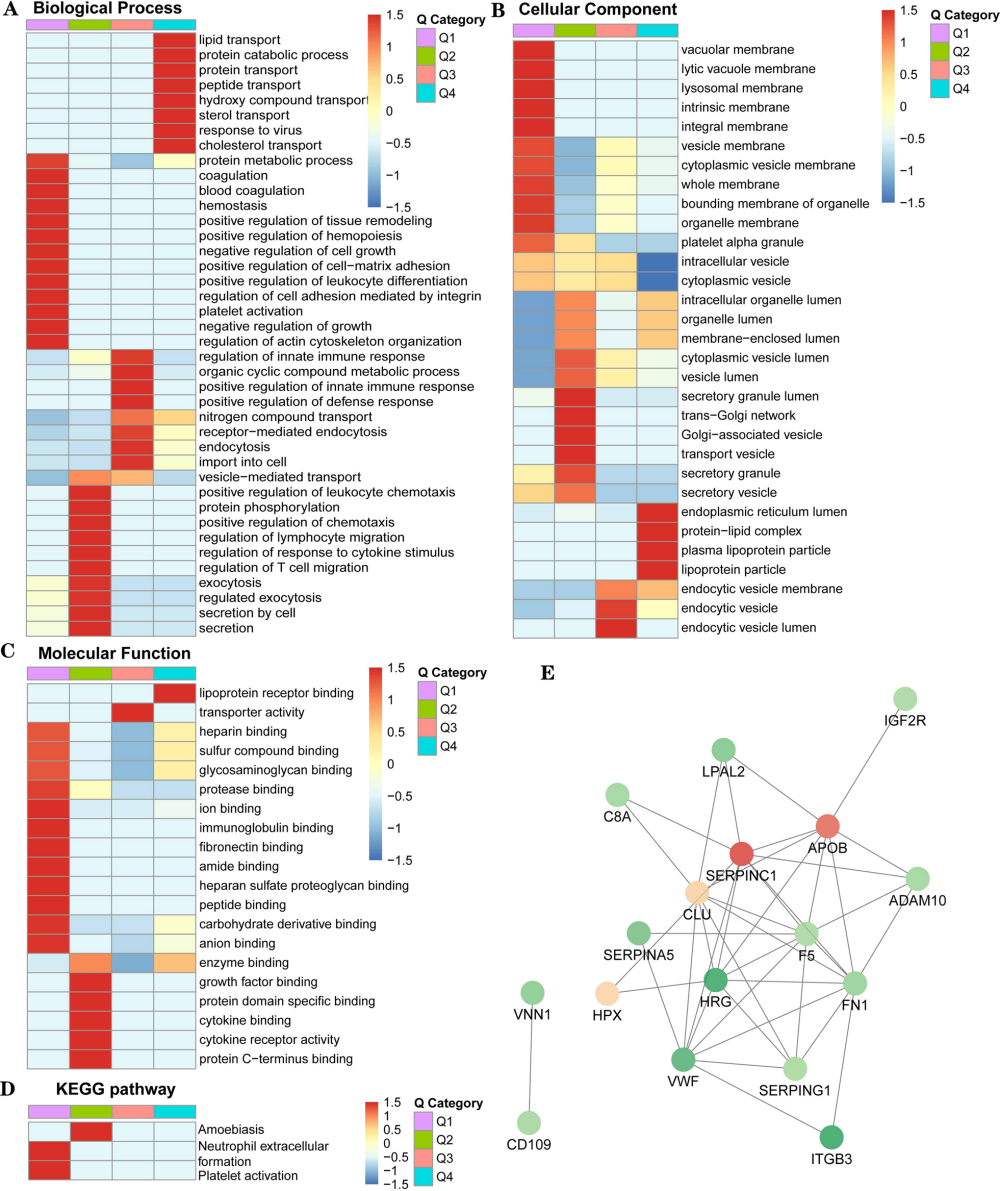
Annotation of differential N-glycosylated proteins in LUAD samples. **A**–**D**. Functional enrichment-based clustering analysis for quantified glycoproteome. **A** biological process analysis, **B** cellular component, **C** molecular function analysis, **D** KEGG pathway analysis. **E** PPI network analysis. Each node represents an N-glycoprotein and each edge represents the interaction between proteins

To elucidate the potential functions of those quantifiable proteins in LUAD samples, we analyzed the quantifiable proteome data set for three enrichment gene ontology (GO) categories: molecular function, cellular compartment and biological process. Based on accumulative normal distribution, we divided all pathways into 4 quantiles: Q1 (< 0.769 fold change), Q2 (0.769–0.833 fold change), Q3 (1.2–1.3 fold change) and Q4 (> 1.3 fold change). In the biological process category, the significant increased pathways enriched in cell migration process such as tissue remodeling, cell growth, cell–matrix adhesion and actin cytoskeleton organization (Q1), and also contained immune-related regulation including leuckocyte chemotaxis, lymphocyte migration, cytokine stimulus and T cell migration (Q2). In Q3 and Q4 analysis, we also found that activated pathways in LUAD samples aggregated in innate immune response, endocytosis (Q3) and in metabolic transport process including protein, lipid, sterol and cholesterol (Q4) (Fig. 4A). As to cellular component analysis, the results revealed that assembled components in LUAD patients were intrinsic, integral, vesicle, cytoplasmic and organelle membrane (Q1), trans-Golgi network, vesicle transport and secretory granule lumen (Q2), metabolic components (protein-lipid complex, plasma lipoprotein particle and lipoprotein particles) (Q3) and endocytic vesicles (Q4) (Fig. 4B). Finally we analyzed molecular function pathways in LUAD samples, we observed enrichment focused on binding activity, including protease, fibronectin, carbohydrate and sulfur compound (Q1), enzyme, cytokine and growth factor (Q2), as well as transport activity (Q3) and lipoprotein receptor binding (Q4) (Fig. 4C). We then analyzed KEGG pathways in LUAD samples and found that the enrichment contained neutrophil extracellular formation, platelet activation (Q1) and amoebiasis (Q2) (Fig. 4D).

Finally STRING protein–protein interaction (PPI) dataset was performed to analyze the protein–protein interactions in identified proteins. The results indicated that APOB, SERPINC1 and CLU were the core nodes in interaction network, which interacted with multiple proteins such as IGF2R, C8A, HPX, HRG and ADAM10. FN1 and SERPING1 also acted as central connection nodes, which interacted with SERPINA5, ITGB3, VWF and F5 (Fig. 4E). Taken together, our results indicated that by TMT labeling and LC–MS/MS sequencing, we identified multiple differential proteins and N-glycosylation sites in LUAD patients, which harbored multiple pathways in tumor-related abnormal metabolism and protein transport.

### The role of N-glycosylation sites in diagnosis of LUAD

**Fig. 5:**
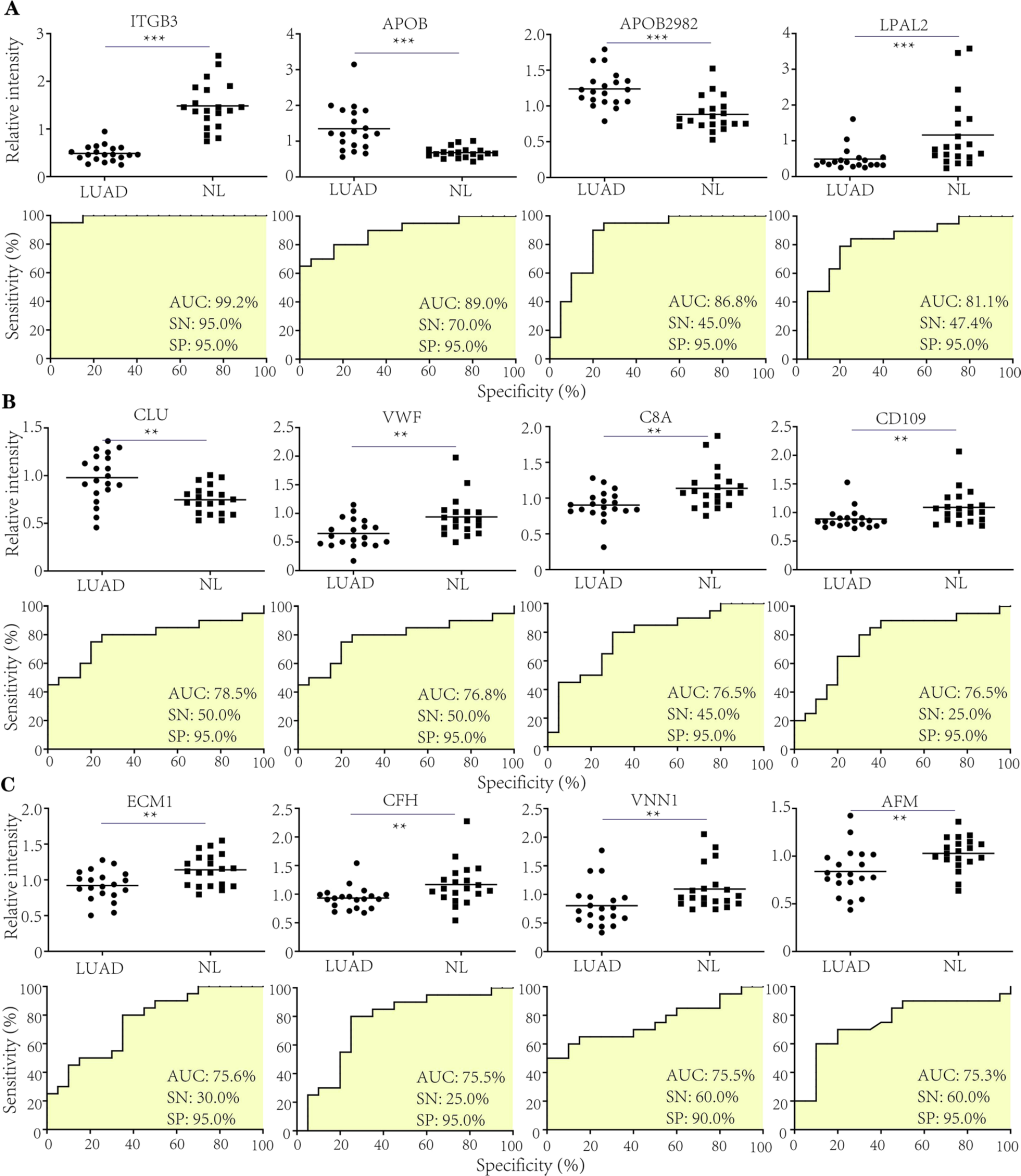
The concentration and ROC analysis of differential N-glycosylation sites identified in LUAD patients. **A** The concentration and ROC analysis of N-glycosylation sites with AUC > 80.0%. **B**, **C** The concentration and ROC analysis of N-glycosylation sites with AUC > 70.0%. **p < 0.01, ***p < 0.001

To further explore the diagnostic accuracy of candidate biomarkers in LUAD, we performed ROC analysis to define the sensitivity (SN) and specificity (SP) of identified N-glycosylation sites. The results suggested that multiple N-glycosylation sites in proteins harbored valuable roles in lung cancer diagnosis. The tope 4 proteins were ITGB3-680, APOB-1523, APOB-2982 and LPAL2-101, which all showed AUC (area under curve) > 80.0% (Fig. 5A). The most important site was ITGB3-680, the AUC was 99.2%, SN (sensitivity) and SP (specificity) were both 95.0% in compared with NL group. In APOB-1523 analysis, the AUC was 89.0%, SN and SP were 70.0% and 95/0%, respectively. In APOB-2982 analysis, AUC was 86.8%, SN was 45.0% and SP was 95.0% when compared with NL group. The AUC in LPAL2-101 analysis was 81.1%, while SN was 47.4%, SP was 95.0% (Fig. 5A).

Besides top 4 proteins, we also analyzed other candidate proteins. We found that AUC of CLU-291 was 78.5%, SN was 50.0% and SP was 95.0%. And the AUC, SN, SP of VWF-2357 were 76.8%, 50.0% and 95.0%, respectively. In C8A-437 analysis, the AUC was 76.5%, SN was 45.0%, SP was 95.0%. In CD109-247 analysis, we obtained AUC was 76.5%, SN was 25.0% and SP was 95.0% (Fig. 5B).

Finally we investigate ECM1-444, CFH-882, VNN1-283 and AFM-33. The analysis results indicated that in ECM1-444, the AUC was 75.6%, SN was 30.0% and SP was 95.0%. In CFH-882, the AUC was 75.5%, SN was 25.0%, SP was 95.0%. In VNN1-283 analysis, the AUC was 75.5%, SN was 60.0% and SP was 90.0%. In AFM-33 analysis, the result showed that AUC was 75.3%, while SN and SP were 60.0% and 95.0%, respectively (Fig. 5C).

In summary, our study revealed multiple N-glycosylation sites harbored highly potential diagnostic value in LUAD diagnosis.

### Combination analysis of novel N-glycosylation sites by machine learning model

**Fig. 6:**
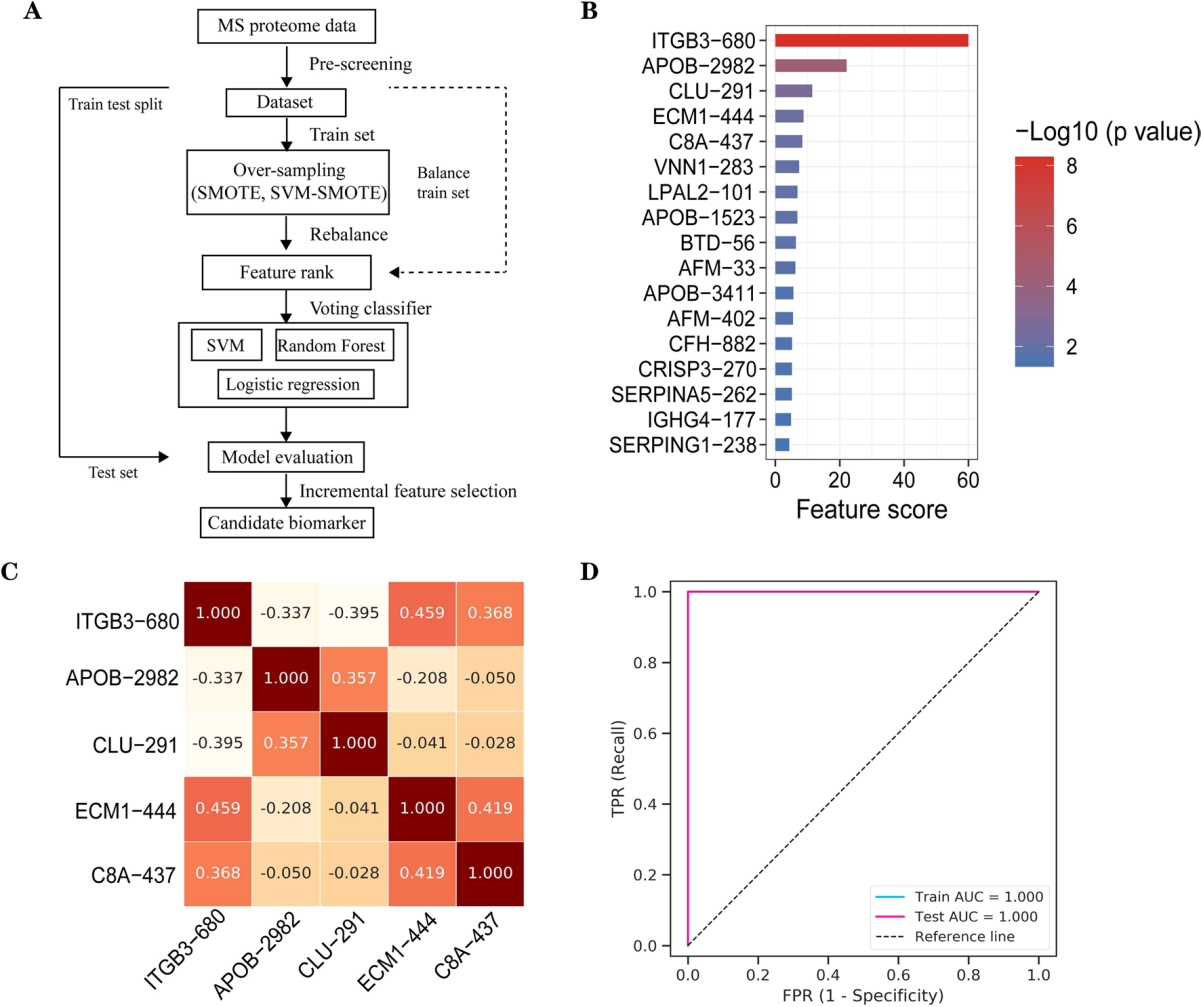
Combination analysis of candidate biomarkers by machine learning. **A** Analysis schema of machine learning. **B** Feature score of candidate N-glycosylation sites identified by feature selection. **C** Pearson correlation coefficient in Top 5 N-glycosylation sites in feature score. **D** AUC in training (16 cases) and test set (4 cases) by plotting the true positive rate against the false positive rate under different cut-off values

IN this part, we introduced machine learning model to test the diagnosis efficiency of candidate biomarkers in lung cancer. We divided all participants into training set (establish model and adjust parameters, 16 cases) and test set (evaluate the model, 4 cases). By combining feature selection, machine learning algorithm, classifier integration method and dataset validation, random forest model was conducted to determine whether the proteomic profile had cancer-specific features for lung cancer diagnosis (Fig. 6A). Due to small sample sizes of two data sets, 2/3 individuals in the training set were selected to grow decision trees by boostrapping and the remaining participants were used as out of bag samples for cross-validation importance. In feature selection, each sample was represented by feature vector, which contained 24 expression features and each expression feature has different ability to distinguish different types of samples. Univariate feature analysis was introduced to quantify the ability of expression features in distinguishing different samples and we could calculate the correlation between each feature and sample types by variance test. Based on this method, the feature scores and p value of candidate molecules revealed the top 15 molecules in candidate N-glycosylation sites, including ITGB3-680, APOB-2982, CLU-291, ECM1-444, C8A-437, VNN1-283, LPAL2-101, APOB-1523, BTD-56, AFM-33, APOB-3411, AFM-402, CFH-882, CRISP3-270, SERPINA5-262, IGHG4-177 and SERPING1-238, which were performed for model construction (Fig. 6B). Next, we analyzed the Pearson correlation coefficient to understand the linear correlation of top 5 proteins (ITGB3-680, APOB-2982, CLU-291, ECM1-444 and C8A-437), the results indicated that ITGB3-680, ECM1-444 and C8A-437 correlated closely (0.459 and 0.368) while APOB-2982 and CLU-291 had correlation (0.357) (Fig. 6C). Finally machine learning model was established to evaluate the role of candidate biomarkers in lung cancer diagnosis. In this study we used logistic regression, support vector machine and random forest as base classifier to construct voting classifier. To evaluate the differences between prediction and actual category, four calculation accuracy index, including sensitivity, specificity, Mattthews’ correlation coefficient and AUC (area under curve) were introduced in this model. In all expression features, the optimal expression feature subset in current data set was selected to obtain optimal prediction accuracy by incremental feature selection (IFS). The AUC curves in training and test set were obtained by plotting the true positive rate against the false positive rate under different cut-off values, and the result indicated that AUC reached 100% in both training and test sets (Fig. 6D). In summary, the machine learning model revealed that combination of N-glycosylation sites had important application in lung cancer diagnosis.

## Discussion

Lack of effectively early diagnostic strategies is the main reason that most lung cancer patients are diagnosed at advanced stages. Thus, it is important to screen and identify valuable biomarkers for early diagnosis of lung cancer. Nowadays, multiple novel circulating biomarkers had valuable potential in lung cancer diagnosis, including circulating tumor DNA (ctDNA), circulating tumor cells, auto-antibodies and exosomes. N-linked glycosylation is a post-translational modifications in cancer progression, which was important in numerous regulations in disease pathogenesis, protein folding, receptor-ligand interactions and tumor-specific immune responses [22]. Increased evidences confirmed the importance of N-glycosylation in molecular structure formation and biological networks in human cells. About 50% of human proteins, either secreting or membrane proteins, harbor N-linked glycosylation patterns, which affect various aspects of biological behaviors in malignant tumor cells [23]. Based on the fact that altered glycosylation levels are hallmarks of tumor progression, circulating tumor-specific glycoproteins can act as candidate novel biomarkers in tumor diagnosis [24]. In small cell lung cancer (SCLC) study, label-free proteomics and multiple reaction monitoring-mass spectrometry were conducted to screen sera from 54 SCLC patients and 29 health controls, the assay revealed four fucosylated proteins APCS, C9, SERPINA4 and PON1, APCS showed 87.5% AUC and PON1 exhibited 91% AUC in extensive stage of SCLC [25]. In another research, fucosylated glycoproteins carbohydrate antigen 19–1 and a-fetoprotein (AFP)-L3 exhibited potential diagnosis role in sera from pancreatic and liver cancer patients [26]. However, the role of N-glycosylation sites in early diagnosis of malignant tumor still needs extensive investigation.

In this study, we enrolled 40 clinical plasma samples, including 20 LUAD and 20 NL to screening tumor specific N-glycosylation sites. TMT-labeling and LC–MS/MS were conducted to investigate plasma protein profiles and identify differentiation proteins, as well as differential N-glycosylation sites between LUAD and NL. Our detection uncovered total 31 differential proteins and 39 differential N-glycosylation sites in LUAD samples, 17 were up-regulated and 22 were down-regulated. Extensive analysis revealed 39 sites harbored potential value in future clinical application. Among all candidate molecules, the most important site was ITGB3-680, which showed highest AUC (99.2%), SN (95.0%) and SP (95.0%) in all proteins. Besides, APOB-1523 (AUC: 89.0%), APOB-2982 (AUC: 86.8%) and LPAL2-101 (AUC: 81.1%) were also important in diagnosis of lung cancer. Due to limitation of single molecule in tumor diagnosis, we then conducted machine learning model to evaluate the combination of candidate N-glycosylation sites. We divided all samples into training and testing groups and the result indicated that both groups harbored 100% AUC, which suggested that these N-glycosylation sites could act as potential biomarkers in application of early diagnosis of LUAD.

In all proteins identified in our study, ITGB3 belonged to integrin family, which enrolled in stress resistance and promoted metastases of triple-negative breast cancer, and mechanism analysis indicated that ITGB3 played a central role in endocytosis of extracellular vesicles by interacting and activating focal adhesion kinase (FAK) [27]. APOB was the main low-density lipoprotein and related closely to poor prognosis of multiple malignant tumors, five independent single nucleotide polymorphism (SNPs) sites significantly associated with NSCLC survival in both discovery and validation datasets [28]. LPAL2 was lipoprotein (A) like 2, which was a pseudogene and contributed to tumor metastasis, lncRNA of LPAL2 could be applied as biomarkers in malignant cholangiocytes [29]. CLU (clusterin) was involved in tumor progression by promoting C-Myc transcriptional repression and immune response, the microarray method and weighted expression profile revealed that decrease CLU could predict the poor survival of lung cancer [30]. ECM1 (extracellular matrix protein 1) was glycoprotein and promoted tumor progression by regulating variety of biological processes such as cell mineralization, proliferation, migration and angiogenesis, the study in gastric cancer revealed that ECM1 could enhance glucose metabolism by inducing FAK/SOX2 signaling pathway [31]. CD109 was a glycosyl phosphatidylinositol anchored protein and enhanced expression of CD109 could be found in multiple tumors including lung cancer, glioblastomas, melanomas and breast carcinoma [32]. CFH was a complement factor and high mRNA level related closely to progression of cutaneous squamous cell carcinoma [33]. In our study, N-glycosylation of these proteins were all important in diagnosis of lung adenocarcinoma.

However, our study also had several shortcomings. Firstly, this was a small cohort which contained only 40 participants (20 tumor patients and 20 normal controls). The second was that we only enrolled LUAD patients and NL controls. Thirdly, our study lacked of molecular mechanism of whether N-glycosylation sites could affect the biological functions of lung cancer. In our next study, we should contain benign lung diseases, including harmartoma, atypical hyperplasia, inflammatory pseudotumor, inflammatory nodules and infections, as well as lung squamous carcinoma (LUSC) and enlarged participants (at least 150 individuals in each group). Moreover, extensive research also needed to investigate the regulation mechanism of N-glycosylation sites in lung cancer biological functions.

In summary, our study provided a primary investigation to screening N-glycosylation biomarkers for early diagnosis of lung cancer. Our study indicated that based on proteomic profiling, N-glycosylation sites derived from plasma could be applied as non-invasive biomarkers for early diagnosis of lung cancer. The combination of these N-glycosylation could significantly increase specificity and sensitivity in diagnosis of lung cancer.

## Supplementary Information

**Additional file 1: Table S1.** MS identified information in this study.

## Data Availability

Data are available via ProteomeXchange with identifier PXD036198.

## Abbreviations

NSCLC: Non-small cell lung cancer
SCLC: Small cell lung cancer
LUAD: Lung adenocarcinoma
LUSC: Lung squamous carcinoma
NL: Normal healthy contrl
MS: Mass spectrometry
CT: Computed tomography
CEA: Carcinoembryonic antigen
CA125: Carbohydrate antigen 12-5
CA19-9: Carbohydrate antigen 19-9
NSE: Neuron specific enolase
CTC: Circulating tumor cells
AAbs: Autoantibodies
KEGG: Kyoto Encyclopedia of Genes and Genomes
ITGB3: Integrin subunit beta 3
APOB: Apolipoprotein B
LPAL2: Lipoprotein (A) like 2
CLU: Clusterin
ECM1: Extracellular matrix protein 1
CFH: Complement factor H
